# Effects of chemoradiotherapy on surface PD-L1 expression in esophageal cancer and its implications for immunotherapy

**DOI:** 10.3389/fimmu.2024.1509051

**Published:** 2024-12-23

**Authors:** Lovis Hampe, Stefan Küffer, Tim Niemeier, Niklas Christian Scheele, Laetitia Zoe Hampe, Anna Luisa Riedl, Laura Anna Fischer, David Alexander Ziegler, Martin Leu, Leif Hendrik Dröge, Alexander König, Michael Ghadimi, Friederike Braulke, Stefan Rieken, Hanibal Bohnenberger, Rami A. El Shafie

**Affiliations:** ^1^ Translational Radiobiology Lab, Department of Radiotherapy and Radiation Oncology, University Medical Center Göttingen, Göttingen, Germany; ^2^ Institute of Pathology, University Medical Center Göttingen, Göttingen, Germany; ^3^ Department of General, Visceral and Thoracic Surgery, University Hospital, Hamburg, Germany; ^4^ Department of Gastroenterology and Gastrointestinal Oncology, University Medical Center, Göttingen, Germany; ^5^ Department of General, Visceral and Pediatric Surgery, University Medical Center, Göttingen, Germany; ^6^ Göttingen Comprehensive Cancer Center (G-CCC), University Medical Center Göttingen, Göttingen, Germany

**Keywords:** esophageal cancer, immunotherapy, programmed-death-ligand-1, checkmate-577, CROSS

## Abstract

**Background:**

Esophageal cancer has a poor prognosis despite treatment advancements. Although the benefit of neoadjuvant chemoradiotherapy (CRT) followed by adjuvant immunotherapy is evident, the effects of CRT on PD-L1 expression in esophageal cancer are not well understood. This study examines the impact of neoadjuvant CRT on PD-L1 surface expression in esophageal cancer both *in vitro* and *in vivo* considering its implications for immunotherapy.

**Methods:**

PD-L1 expression dynamics were assessed in esophageal adenocarcinoma (EAC) and esophageal squamous cell carcinoma (ESCC) cell lines (OE-33, FLO-1, KYSE-180) treated with Carboplatin, Paclitaxel, radiotherapy (RT), and CRT. PD-L1 expression was measured by flow cytometry at 48- and 72 hours post-treatment. Temporal changes of surface PD-L1 were further investigated in KYSE-180 cells following RT, up to 168h after treatment. Additionally, PD-L1 expression was analyzed via immunohistochemistry in histological samples from 19 patients (9 EAC, 10 ESCC) treated with neoadjuvant CRT according to the CROSS-scheme.

**Results:**

PD-L1 expression was upregulated the most by Carboplatin, a combination of chemotherapy, or CRT in all cell lines. Higher irradiation doses were more effective in inducing PD-L1 expression, while Paclitaxel alone did not consistently increase PD-L1. The ESCC cell line KYSE-180 showed the highest relative PD-L1 increase. Measurement of PD-L1 kinetics revealed a transient upregulation of surface PD-L1, which peaked at 72 hours post-treatment and subsequently returned to baseline levels by 168 hours. *In vivo*, data demonstrated no significant PD-L1 expression changes when comparing pre- and post-treatment levels.

**Conclusions:**

Chemotherapy, RT, and CRT can induce PD-L1 expression in various esophageal cancer cell lines. However, neoadjuvant CRT according to the CROSS protocol does not significantly induce PD-L1 *in vivo*. Considering the difference in time between pre- and post-therapeutic measurements, these findings suggest that PD-L1 upregulation due to neoadjuvant therapy may be transient *in vivo* as well. This highlights the potential benefit of administering immunotherapy in a neoadjuvant setting.

## 1 Introduction

Esophageal cancer, currently the eighth most common cancer globally, remains the sixth leading cause of cancer-related deaths (1). The incidence of this malignancy is anticipated to rise in the forthcoming years, reflecting a continued upward trend observed over time (2). The prognosis of esophageal cancer remains poor with a 5-year-survival rate of only 18-20 percent throughout all stages (3, 4). Survival rates are notably lower when the disease is diagnosed at locally advanced or metastatic stages (1). For such cases, neoadjuvant chemoradiotherapy (CRT) using Carboplatin and Paclitaxel, combined with radiotherapy (RT) at a cumulative dose of 41.4 Gy in 23 fractions (known as the CROSS regimen), has been shown to offer clinical benefits (5). More recent studies have indicated that patients who receive neoadjuvant CRT followed by adjuvant nivolumab in the presence of residual tumor experience improved disease-free survival (DFS), irrespective of their pretherapeutic PD-L1 combined positive score (CPS) (6).

PD-L1 expression on the surface of tumor cells is a recognized prognostic factor for response to immunotherapy (7). Although evidence from various tumor types suggests that PD-L1 expression is upregulated in response to CRT and its modalities (8–10), similar evidence specifically addressing the effects of radiotherapy and chemoradiotherapy on PD-L1 surface expression in esophageal cancer remains limited (8, 11). Moreover, real-world data regarding differences in pre- and post-therapeutic PD-L1 expression in esophageal cancer patients is lacking. It is plausible that an increase in surface PD-L1 following neoadjuvant CRT could enhance the efficacy of subsequent adjuvant immunotherapy, particularly in patients with low pretherapeutic PD-L1 scores.

Recent clinical research has focused on optimizing the timing of immunotherapy in esophageal cancer (12). The existing literature regarding other tumor entities increasingly suggests a potential benefit associated with the use of neoadjuvant immunotherapy (13–15). However, there is a notable gap in the literature regarding the kinetics of potential PD-L1-enhancing effects associated with neoadjuvant therapy in real patients. Further investigation of these dynamics could significantly contribute to a deeper understanding of the potential advantages of neoadjuvant immunotherapy.

In this study, we sought to further investigate the changes in PD-L1 surface expression in response to various therapeutic modalities of neoadjuvant CRT administered according to the CROSS protocol. This included analyzing the effects of radiotherapy, chemotherapy (CT), and their combination on esophageal adenocarcinoma EAC and ESCC. The investigation was conducted both *in vitro*, using the cell lines FLO-1, OE-33, and KYSE-180, and *in vivo*, by examining histologic slides from patients treated with neoadjuvant CROSS CRT.

## 2 Material and methods

### 2.1 Cell culture

Established EAC cell lines OE-33 with high surface PD-L1 baseline expression (DSMZ, Germany) and FLO-1 with low surface PD-L1 baseline expression (DSMZ, Germany) were used as well as ESCC cell line KYSE-180 with mid surface PD-L1 baseline expression (DSMZ, Germany). All cell lines were provided by German Collection of Microorgamisms and Cell Cultures GmbH (DSMZ). OE-33, FLO-1 and KYSE-180 were cultured in T75 cell culture flasks (Sarstedt, Nümbrecht, Germany) using RPMI 1640 Glutamax^®^ (Gibco, USA) medium, supplemented with 10% heat-inactivated FBS (Serana, Germany). All cell lines were cultured at 37°C/5% CO2 and tested to be free of mycoplasma regularly.

### 2.2 Reagents

Carboplatin (Thermo Fisher Scientific Chemicals) was dissolved in water at a stock concentration of 10,000µM and stored at 2°C. Paclitaxel (Thermo Fisher Scientific Chemicals, USA) was dissolved in <0.1% dimethylsulfoxide (Sigma Aldrich, USA) at a stock concentration of 10,000nM while stored at -20°C. All chemotherapeutic agents were diluted in RPMI 1640 Glutamax^®^ media (Gibco, USA) before treatment. A detailed list of all reagents is provided in the (**Supplementary Materials S14**).

### 2.3 Viability assays

Cells were seeded in 96-well-plates (Greiner, Austria) at a density of 2,500 cells of KYSE-180/OE-33 (25 cells/µl) and 7,500 cells of FLO-1 (75 cells/µl). After attachment of the cells overnight, cells were treated in ascending concentrations of Carboplatin (5µM, 10µM, 20µM, 50µM, 100µM, 200µM, 500µM, 1000µM, 2000µM) or Paclitaxel (2nM, 5nM, 10nM, 20nM, 50nM, 100nM, 200nM, 2000nM) in a total volume of 100µl. After treatment for 72 hours, cell viability was tested adding 20µl of CellTiter 96^®^ Aqueous One Solution Cell Proliferation Assay (Promega, USA). All experiments included five technical replicates and were repeated three times. IC50 was calculated via nonlinear regression using Prism 10 (GraphPad, USA).

For IC50 determination of combination treatment, SynergyFinder 3.0 was used (16). Cells were seeded in a 6x6 scheme at the density and volume stated above and treated with ascending concentrations of Carboplatin (5µM, 10µM, 25µM, 50µM, 100µM) and Paclitaxel (1nM, 2.5nM, 5nM, 10nM, 25nM) on day two. Cell viability was tested adding 20µl of CellTiter 96^®^ Aqueous One Solution Cell Proliferation Assay (MTS). The experiments were replicated seven times.

IC50 of chemoradiotherapy was determined using the same MTS-protocol with 10,000µM of Carboplatin as negative control and media as positive control.

### 2.4 Treatment of tumor cell lines

For treatment up to 72h, cells were seeded at 500,000 cells in T75 cell culture flasks (Sarstedt, Nümbrecht, Germany) in a volume of 15ml of media (33,333 cells/ml) and treated on day two. In case of measurement after 168h, cells were seeded at 50,000 cells in T75 cell culture flasks (Sarstedt, Nümbrecht, Germany). Chemotherapeutic agents were diluted in media in respective concentrations. Cells were irradiated using an X-ray generator (225 kV, 17.6 mA; Kubtec Scientific, Germany). For improved comparability between treatment modalities, 50% inhibition was aimed for in Carboplatin and Paclitaxel single drug treatments, combination treatment and chemoradiotherapy, respectively. IC50 calculations for all three cell lines, derived from single-drug treatments with Carboplatin and Paclitaxel (S4–9) and combination treatment (S10–12), are provided in the **Supplementary Materials**.

### 2.5 Flow cytometry

48 and 72 hours after treatment tumor cells were harvested for analysis. After washing the cells with DPBS (Gibco, USA) twice, 2 – 5 × cells were incubated with Alexa Fluor 700 conjugated anti-PD-L1 antibody (MIH1) (Thermo Fisher Scientific, USA), and the respective isotype control (Thermo Fisher Scientific, USA) diluted in a total of 100µl of FACS-buffer (2,000-5,000 cells/µl) for 45 minutes at 4°C on an orbital shaker. After the incubation period cells were washed with FACS-buffer twice and incubated with Propidium Iodide (Becton Dickson, USA) for 10 minutes at 4°C. Cells then were analyzed with flow cytometry (FACS Celesta^®^, Becton Dickson, USA). Due to immediacy of the protocol, no fixation of cells was performed. Delta mean fluorescence intensity was calculated by subtracting mean fluorescence intensity of anti-PD-L1 samples with respective isotype control samples. FlowJo v10.10 software was used to analyze at least 10,000 events.

### 2.6 Cell block

To validate flow cytometry data, cell blocks of all cell lines were created after CRT and stained via immunohistochemistry. To create cell blocks, cells were treated in T-75 flasks with CRT as stated above and scraped off into 2ml eppendorf cups (Eppendorf SE, Germany). Cells were washed with 4°C PBS and centrifuged at 200g twice. The supernatant was removed, and the cells were fixed with formalin. Treated cell lines were harvested and centrifuged at 300g for 3 minutes at room temperature. The resulting pellets were resuspended in 500 µL of agarose, chilled at 4°C for 20 minutes, and fixed overnight in 4% buffered paraformaldehyde. The solidified agarose cones were then embedded in paraffin. These cell blocks were sectioned into 2-μm slices and incubated in EnVision Flex Target Retrieval Solution (low pH, Dako Agilent). This was followed by incubation with the primary antibody against PD-L1 (22C3 pharmDx, Dako Agilent, ready-to-use) for 20 minutes at room temperature. Subsequently, the sections were treated with the secondary antibody (EnVision Flex+, Dako Agilent), and immunostaining was visualized using DAB (Dako Agilent). Counterstaining was performed with Mayer’s hematoxylin. The samples were analyzed under a light microscope, and the percentage of PD-L1-positive tumor cells was calculated to determine the TPS score.

### 2.7 Patients

Patients with esophageal cancer, treated with neoadjuvant CRT analogous to the CROSS-scheme between 2015 and 2024 at a single tertiary comprehensive cancer center (University Medical Center Göttingen) were reviewed in retrospect. To be included, patients had to have undergone their diagnostic biopsy and therapeutic resection of the esophagus at the center to ensure comprehensive access to the patient material. For PD-L1 staining, patients needed to have >100 residual tumor cells in the resected esophagus. Furthermore, they must have undergone the full neoadjuvant CRT and not discontinued therapy due to adverse events. 19 Patients (9 EAC, 10 ESCC) remained eligible for PD-L1 staining. All scoring was done centrally by a single specialized pathologist (H.B.). The study was approved by the ethics committee of the University Medical Center Göttingen (number 6/9/24).

### 2.8 Statistical analysis

For flow cytometry data, the arithmetic mean of values was calculated using FlowJo v10.10 (Becton Dickson, USA). A one-tailed Mann-Whitney-U-test was used for analyses, performed in Prism 10 (GraphPad, USA). Statistical analysis of patient PD-L1 scores was performed with Prism 10 (GraphPad, USA) using one-tailed Wilcoxon signed rank test. Statistical significance is stated as *p < 0.05; **p < 0.01; ***p < 0.001.

## 3 Results

### 3.1 Surface PD-L1 is increased in tumor cell lines *in vitro*

We assessed the alterations in surface PD-L1 expression in EAC cell lines FLO-1 and OE-33 as well as in ESCC cell line KYSE-180 at 48- and 72-hours following treatment. The treatment regimen was based on the CROSS protocol, comprising carboplatin, paclitaxel, and irradiation (5). We evaluated the effects of individual treatments, combination therapies, and the dose-dependent impact of irradiation on surface PD-L1. To ensure comparability across single-agent CT, combination therapies, and CRT, the treatments were standardized to achieve approximately 50% cell viability post-treatment. This was determined using MTS assays for single drug CT, and SynergyFinder 3.0 (16) for combination treatments (**Supplementary Figures S4**-**12**).

#### 3.1.1 FLO-1

**Figure 1:**
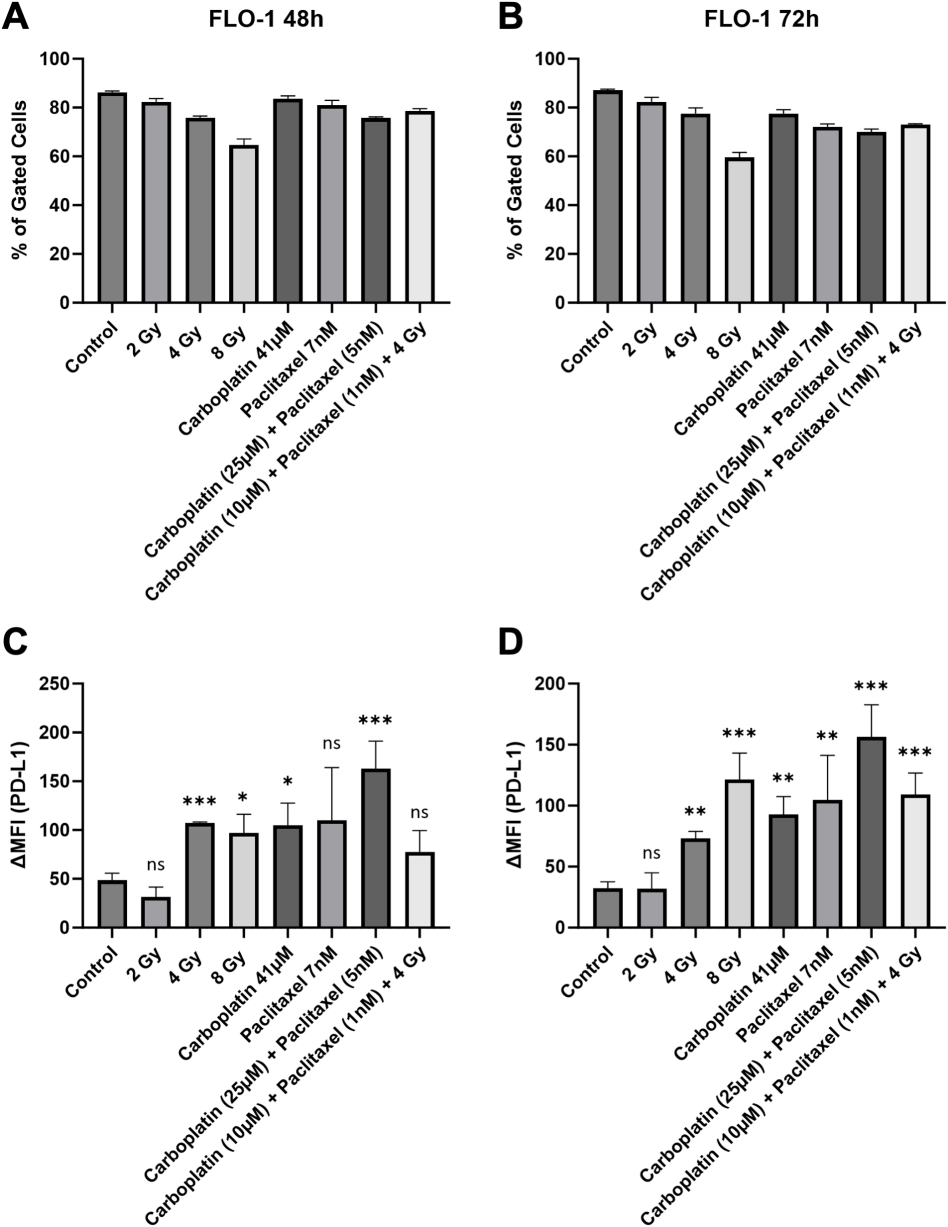
Number of gated cells **(A, B)** and surface PD-L1 expression **(C, D)** were analyzed via flow cytometry in low baseline PD-L1 EAC cell line FLO-1, 48 and 72 hours after treatment. Delta mean fluorescence intensity (ΔMFI) was calculated by subtracting the mean fluorescence intensity of anti-PD-L1 samples from the respective isotype control samples. FlowJo v10.10 software was used to analyze at least 10,000 events. Statistical significance is indicated as follows: *p < 0.05; **p < 0.01; ***p < 0.001, with error bars representing the standard error of the mean (SEM). ns stands for "not significant".

In EAC cell line FLO-1, PD-L1 induction was measured most reliably after 72 hours (**Figure 1D**), while combination treatment with Carboplatin and Paclitaxel, as well as single dose irradiation with 4 Gy and 8 Gy showed the strongest effect in regard to single drug treatment. Carboplatin induced PD-L1 more reliably than Paclitaxel did. Low dose irradiation with 2 Gy had no significant effects on the expression of PD-L1 (**Figures 1C, D**). Gated cells remained consistent at both time points with irradiation at a dose of 8 Gy showing the lowest number of gated cells due to the high cytotoxicity (**Figures 1A, B**). Representative flow cytometry histograms of FLO-1 illustrating the response to therapeutic modalities are provided in the **Supplementary Materials** (**Supplementary Figure S1A**). Due to the relatively low level of PD-L1 even after CRT, the effects visible in the cell block are mild, yet visible (**Supplementary Figure S2**).

#### 3.1.2 KYSE-180

**Figure 2:**
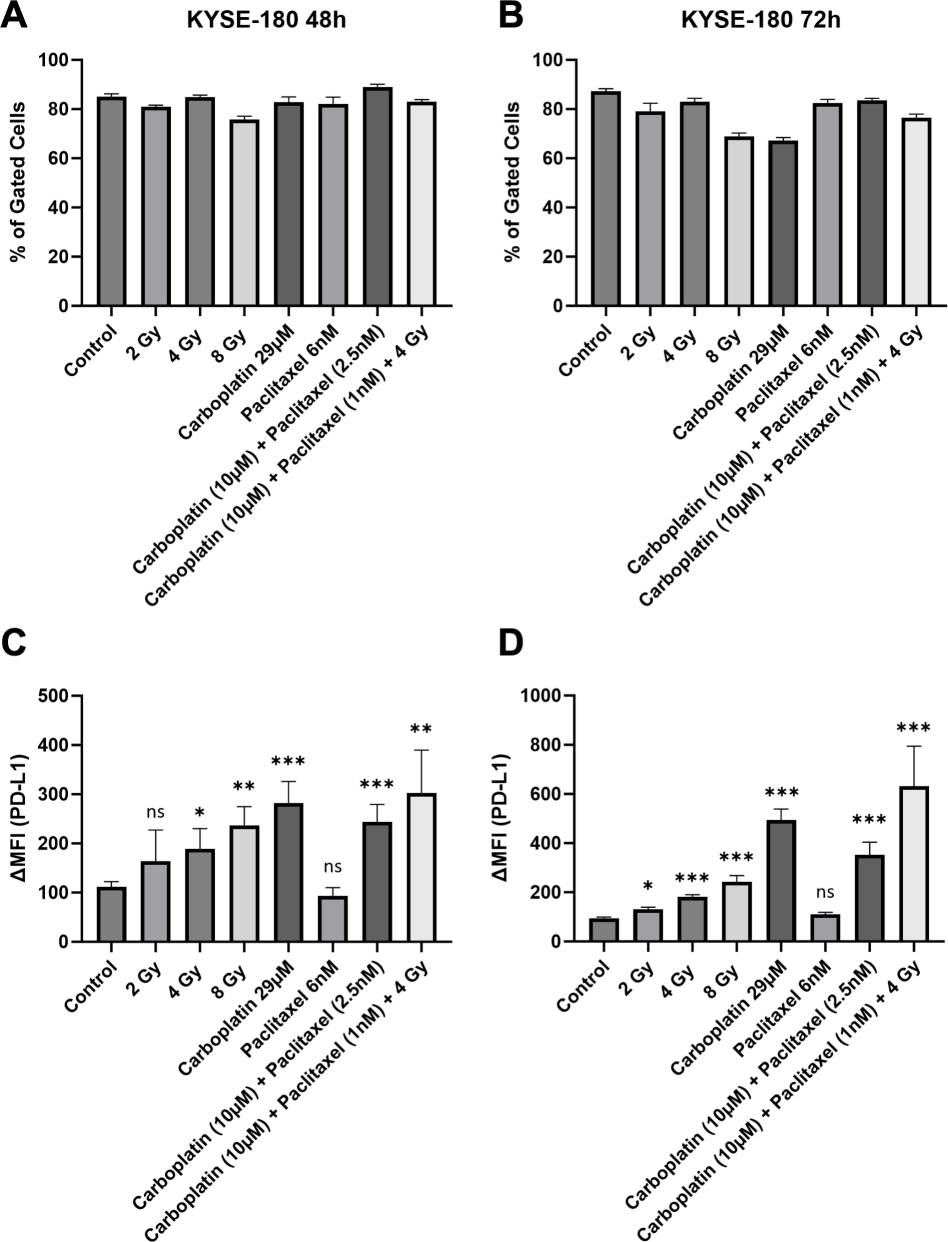
Number of gated cells **(A, B)** and surface PD-L1 expression **(C, D)** were analyzed via flow cytometry in mid baseline PD-L1 ESCC cell line KYSE-180, 48 and 72 hours after treatment. Delta mean fluorescence intensity (ΔMFI) was calculated by subtracting the mean fluorescence intensity of anti-PD-L1 samples from the respective isotype control samples. FlowJo v10.10 software was used to analyze at least 10,000 events. Statistical significance is indicated as follows: *p < 0.05; **p < 0.01; ***p < 0.001, with error bars representing the standard error of the mean (SEM). ns stands for "not significant".

After 72 hours, surface PD-L1 was increased the most in ESCC cell line KYSE-180, compared to 48 hours. Higher doses of irradiation led to a greater response in the expression of PD-L1 (**Figures 2C, D**). At the 72-hour timepoint, KYSE-180 is the only cell line in our experiment, that showed a significant increase in surface PD-L1 after irradiation with 2 Gy (**Figure 2D**). Notably, CRT, combination treatment with Carboplatin and Paclitaxel, and single drug treatment with Carboplatin each induced PD-L1 expression. At both 48-hour and 72-hour time points, single-drug treatment with Paclitaxel did not show significant effects (**Figures 2C, D**). The number of gated cells remained stable at both time points (**Figures 2A, B**).

Overall, ESCC cell line KYSE-180 showed the highest relative increase in PD-L1 when compared to EAC cell lines OE-33 and FLO-1. Representative flow cytometry histograms of KYSE-180 illustrating the response to therapeutic modalities are provided in the **Supplementary Materials** (**Supplementary Figure S1B**).

**Figure 3:**
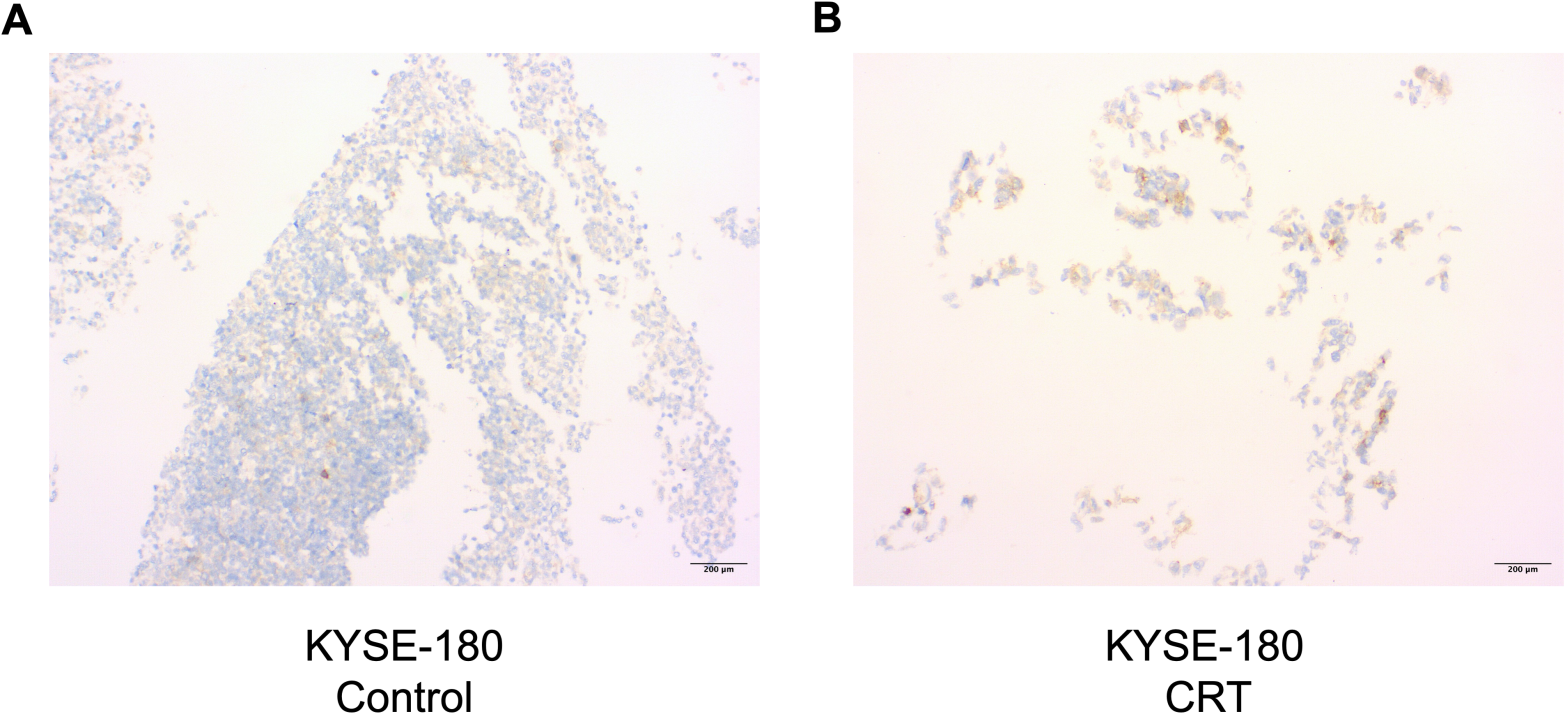
Cell block of KYSE-180 ESCC cell line stained with an anti-PD-L1 antibody via immunohistochemistry at 72-hour timepoint after treatment **(A, B).** Untreated control **(A)** is depicted next to treated sample **(B)** (Carboplatin 10μM, Paclitaxel 1nm, 4Gy). Images are shown at 10x magnification. CRT, chemoradiotherapy.

The effects of chemoradiotherapy (CRT) were prominently observed in the immunohistochemistry analysis. The untreated cell block exhibited a higher cell density and only mild PD-L1 staining (**Figure 3A**). In contrast, the CRT-treated cell block demonstrated a pronounced increase in PD-L1 expression, with a visibly stronger staining intensity (**Figure 3B**).

#### 3.1.3 Surface PD-L1 is increased only transiently

**Figure 4:**
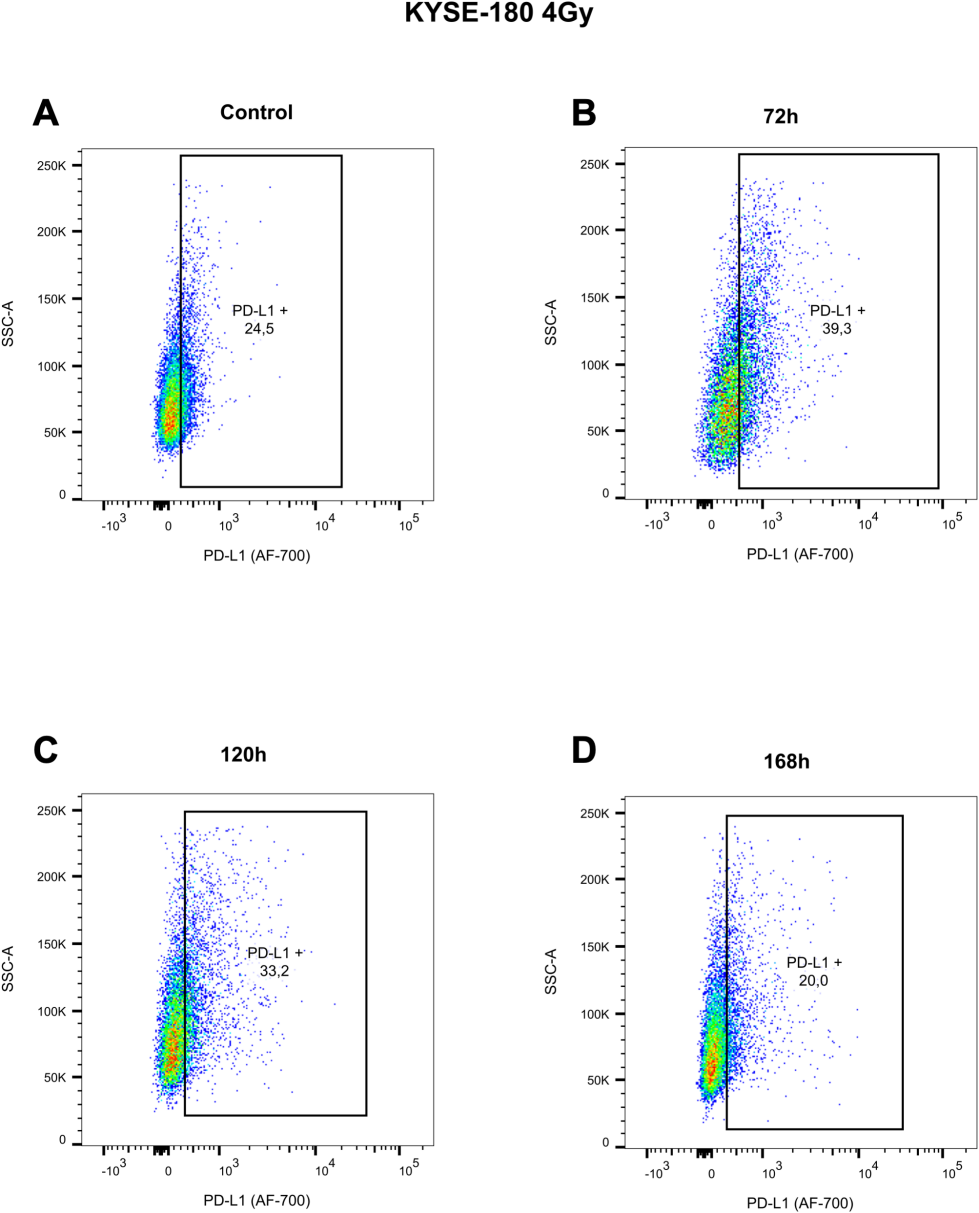
Pseudocolor plots illustrating the dynamic changes in surface PD-L1 expression at 72 hours **(B)**, 120 hours **(C)**, and 168 hours **(D)**, compared to the untreated control **(A)**, following exposure to 4 Gy radiotherapy in the EAC cell line KYSE-180. Gating was performed based on the corresponding isotype control for treatment condition, displayed in **Supplementary Figure 13**.

To determine whether the observed increase in surface PD-L1 expression is transient, the ESCC cell line KYSE-180 was treated with 4 Gy of radiotherapy, and PD-L1 was measured over a period of 168 hours post-treatment (**Figure 4**). Our analysis revealed that PD-L1 expression in KYSE-180 peaks at 72 hours after treatment (**Figure 4B**), followed by a decline at 120 hours (**Figure 4C**) and eventually returns to baseline levels by 168 hours (**Figure 4D**).

#### 3.1.4 OE-33

**Figure 5:**
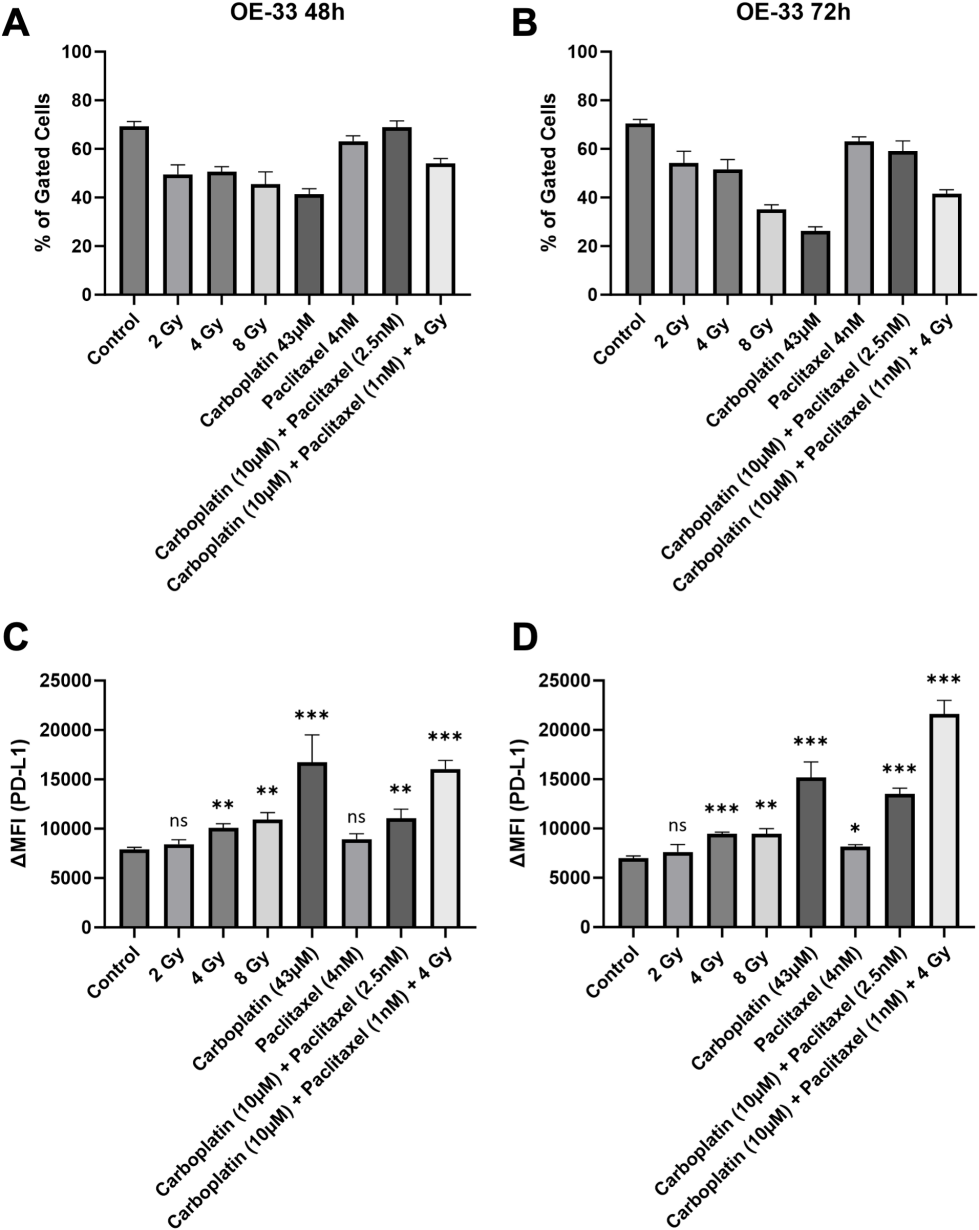
Number of gated cells **(A, B)** and surface PD-L1 expression **(C, D)** were analyzed via flow cytometry in high baseline PD-L1 EAC cell line OE-33, 48 and 72 hours after treatment. Delta mean fluorescence intensity (ΔMFI) was calculated by subtracting the mean fluorescence intensity of anti-PD-L1 samples from the respective isotype control samples. FlowJo v10.10 software was used to analyze at least 10,000 events. Statistical significance is indicated as follows: *p < 0.05; **p < 0.01; ***p < 0.001, with error bars representing the standard error of the mean (SEM). ns stands for "not significant".

EAC cell line OE-33 showed similar responses at the 48-hour and 72-hour time points (**Figures 5C, D**). However, in OE-33 the relative number of gated cells was lowest of all three cell lines in all experimental setups (**Figures 5A, B**). Irradiation doses of 4 Gy, 8 Gy showed similar increases in surface PD-L1. Low dose irradiation of 2 Gy did not induce PD-L1 significantly. Single drug treatment with Carboplatin, combination treatment with Carboplatin and Paclitaxel and CRT were most efficacious in inducing PD-L1 (**Figures 5C, D**). In contrast to the other cell lines used, Paclitaxel treatment of OE-33 showed a significant but small increase in PD-L1 (**Figure 5D**). Representative flow cytometry histograms of KYSE-180 illustrating the response to therapeutic modalities are provided in the **Supplementary Materials** (**Supplementary Figure S1C**). The effects of CRT on surface PD-L1 expression in OE-33 cells could not be discerned through immunohistochemistry, likely due to the inherently high baseline PD-L1 expression in this cell line. Corresponding images of treated and untreated cell blocks are provided in the **Supplementary Materials** (**Supplementary Figure S3**).

### 3.2 Chemoradiation does not consistently induce surface PD-L1 *in vivo*

**Figure 6:**
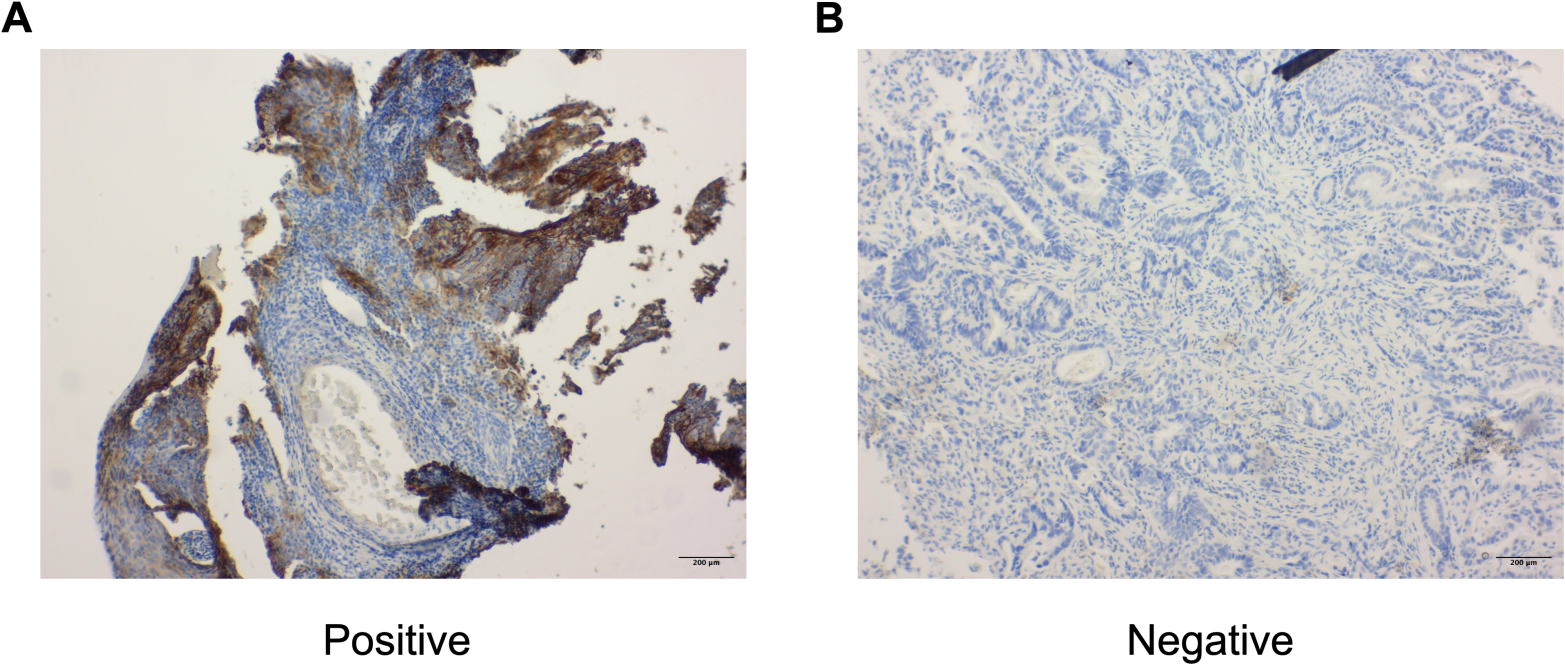
Representative images of immunohistochemical staining with anti-PD-L1 antibody in esophageal cancer patients **(A, B)**. A PD-L1 positive sample **(A)** is shown next to a negative sample **(B)**. All images are shown at 10x magnification.

To further investigate the effects of CRT on surface PD-L1 *in vivo*, we retrospectively selected patients treated with neoadjuvant CROSS CRT followed by esophagectomy at a single tertiary cancer center over a 10 year time period (n=19). We compared the PD-L1 expression of pretherapeutic, diagnostic biopsies with corresponding tumor samples of the resected esophagus using immunohistochemistry. Representative images of immunohistochemical staining are shown in **Figure 6**.

**Figure 7:**
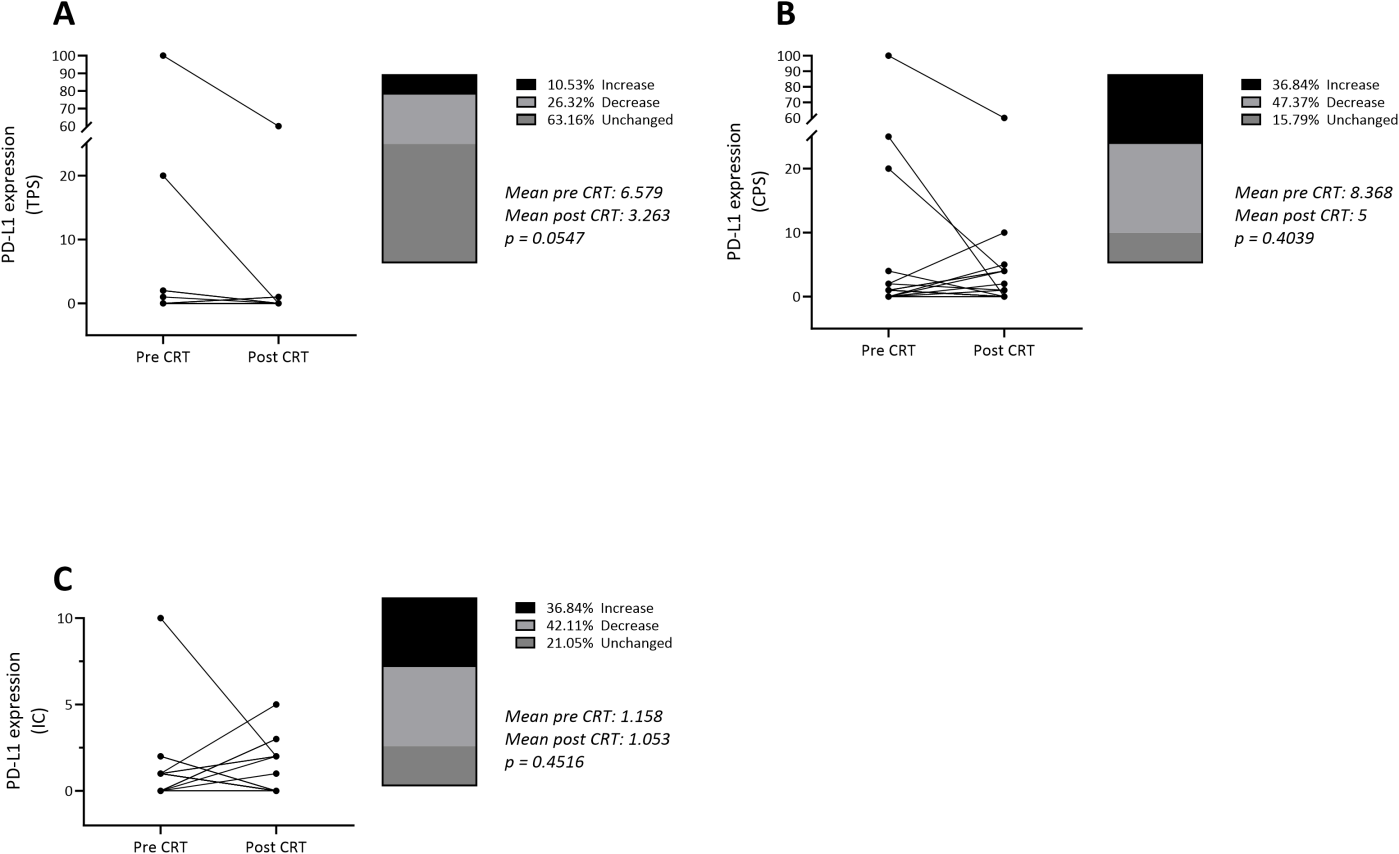
Alteration in PD-L1 surface expression in n = 19 patients treated with neoadjuvant chemoradiotherapy according to CROSS protocol. Scores as stated **(A)** Tumor Proportion Score, **(B)** Combined Positive Score, **(C)** Immune Cells. Wilcoxon signed rank test was used for statistical analysis with statistical significance set as p < 0,05. CRT, chemoradiotherapy.

All patients showed no significant increase in PD-L1 surface expression in response to neoadjuvant CRT. Regarding Tumor Proportion Score (TPS), only 37% of patients (n=7) showed a relevant change, while the proportion of patients with decreasing TPS was higher than the proportion of increasing TPS (26%, n=5 decrease, 11%, n=2 increase) (**Figure 7A**). The alteration of surface PD-L1 in response to CRT was comparable in Combined Positive Score (CPS) and Immune Cells (IC). Both scores showed higher rates in increase than TPS did (37% in CPS and IC). Nevertheless, a higher rate in decreases (47% in CPS, 42% in IC) was seen (**Figures 7B, C**). No differences in response regarding histological subtype were observed.

## 4 Discussion

Neoadjuvant chemoradiotherapy continues to be an important therapeutic strategy for managing locally advanced esophageal cancer (17). A commonly used treatment regimen is the CROSS scheme, which combines preoperative chemoradiotherapy using carboplatin and paclitaxel, followed by surgical resection of the tumor (18). In cases where residual tumor is detected post-resection, additional therapy with PD-1 inhibitors such as nivolumab benefits the patients. Thus the CM577 study demonstrated the efficacy of immunotherapy in improving disease-free survival (DFS) in patients with esophageal cancer, irrespective of their pre-treatment PD-L1 expression levels (6).

Given that PD-L1 expression serves as a predictive biomarker for the response to immunotherapy (7) and preexisting data indicate PD-L1 inducing effects of neoadjuvant CRT in other tumor entities (8), it is plausible that neoadjuvant chemoradiotherapy could increase surface PD-L1 expression and thereby improve clinical outcomes. To investigate this hypothesis, we conducted a study to assess the potential effects of neoadjuvant CRT, as per the CROSS scheme, on PD-L1 expression in ESCC and EAC. Our findings suggest that surface PD-L1 expression is induced by CT, RT, and CRT. Within the CROSS scheme, platinum-based chemotherapy appeared to have the most substantial impact on surface PD-L1 expression. This effect was further amplified when combined with paclitaxel or as a part of the chemoradiotherapy regimen. The effects of platinum-based chemotherapy align with preexisting data from other tumor entities (9, 19, 20).

However, our results show that paclitaxel alone did not reliably induce surface PD-L1 expression, with significant effects observed only in the OE-33 cell line at 72 hours and in the FLO-1 cell line at 48- and 72-hours post-treatment. The available literature indicates that paclitaxel induces PD-L1 expression when used in combination with other therapeutic agents (11), with no existing data on its effects as a monotherapy in esophageal cancer. Notably, paclitaxel has been shown to induce surface PD-L1 expression as a standalone treatment in ovarian cancer (21), whereas data in breast cancer cells remains inconclusive (22). Based on its effects as monotherapy on certain cell lines and its impact in the context of CRT or combination treatments, we hypothesize that paclitaxel is a weak inducer of PD-L1 expression.

Regarding histological subtypes, our *in vitro* data suggests that ESCC exhibits the highest relative increase in surface PD-L1 expression following CRT and may therefore derive the greatest benefit from subsequent immunotherapy. Although there is currently no additional experimental data available on the differences in PD-L1 expression increase between histological subtypes, clinical evidence appears to support this hypothesis. In both the Checkmate 577 study (CM577) and the KEYNOTE-181 trial, patients with ESCC demonstrated a greater benefit from immunotherapy compared to patients with EAC (6, 23).

Our *in vivo* analysis has shown that tumor PD-L1 scores TPS, CPS and IC did not exhibit a consistent pattern of change following neoadjuvant CRT, with most cases showing either unchanged or decreased scores. While these results may seem contradictory to our *in vitro* findings, which demonstrate an increase in PD-L1 following treatment, they are consistent with our *in vitro* data indicating that this observed elevation is only transient. It is important to note that sustained alterations in PD-L1 expression are necessary to influence postoperative PD-L1 assessment effectively. Most of our *in vitro* measurements of PD-L1 expression were conducted up to 72 hours post-treatment, whereas the interval from neoadjuvant CRT to esophagectomy spans the scale of months (24). This temporal discrepancy may explain the challenges in detecting the effects of CRT on surface PD-L1 *in vivo*, which might only be observable through mid-therapeutic biopsies (25). Notably, in the EAC cell line FLO-1, upregulation of PD-L1 was observed to diminish over time, as demonstrated by the comparison between the 48-hour and 72-hour time points.

Therefore, our findings indicate that even though surface PD-L1 expression may be elevated during CRT, CT, or RT, effects are transient, as preexisting literature in other tumor entities indicates (22). This observation could explain the CM577 *post hoc* analysis. Patients with a combined positive score (CPS) of ≤ 5 did not derive statistically significant, yet low, benefit in DFS from adjuvant nivolumab after undergoing neoadjuvant CRT (HR = 0.89, 95% CI: 0.65–1.22) (26). The subpopulation of patients with low pretherapeutic PD-L1 expression would likely benefit the most from an increase in surface PD-L1 induced by neoadjuvant therapy, as this would render them more suitable candidates for immunotherapy (27). It is plausible that any potential clinically relevant increase in PD-L1 expression induced by the neoadjuvant treatment may mostly have declined by the time of esophagectomy and consequently these patients have not benefitted from immunotherapy in an adjuvant setting. Thus, combining neoadjuvant CRT with neoadjuvant immunotherapy could potentially make better use of PD-L1-inducing effects of CRT and represents a promising avenue for future research. Recent studies have already shown benefits in achieving pathological complete remission when employing neoadjuvant immunotherapy and CRT compared to neoadjuvant CRT alone (28). Further research is necessary to compare the efficacy of adjuvant versus neoadjuvant immunotherapy in patients with esophageal cancer treated with neoadjuvant CRT. Furthermore, a pre-therapeutic assessment of PD-L1 expression, combined with a mid-therapeutic biopsy during CRT, could facilitate the evaluation of treatment-induced upregulation of surface PD-L1. In cases where PD-L1 levels demonstrate a significant increase from initially low pre-therapeutic levels, neoadjuvant immunotherapy could harness this transient elevation best. Conversely, cases characterized by high baseline PD-L1 expression may also benefit from immunotherapy administered in an adjuvant setting.

Moreover, research in lung cancer indicates that it is not clear whether stained biopsy samples match the same tumor area as surgically resected samples and therefore correlate within their immunogenic status. Although staining multiple biopsies and surgically resected samples could be a viable strategy (29, 30), this approach may be especially challenging in subpopulations with high response rates to neoadjuvant treatment. Such high response rates result in minimal residual tumor areas, making it difficult to obtain the required minimum of 100 tumor cells for analysis (31). That being said, the selection criteria for our patient cohort exclusively included individuals who did not achieve pathological complete remission (pCR) following neoadjuvant chemoradiotherapy (CRT). Research in breast cancer indicates that high PD-L1 expression serves as a predictive biomarker for achieving pathological complete remission (pCR) following neoadjuvant chemotherapy (32). Therefore, it is reasonable to hypothesize that patients who experienced an upregulation of PD-L1 in response to neoadjuvant CRT may have been excluded from our study due to their higher likelihood of achieving pCR.

## 5 Conclusions

The expression of PD-L1 arose as an important biomarker for immunotherapy in esophageal cancer. However, it is not yet clear whether neoadjuvant chemoradiotherapy effectively induces surface PD-L1 expression in this context, nor how such an effect could be harnessed clinically. Our findings indicate that although PD-L1 expression is increased *in vitro* following neoadjuvant CRT, the effects are only transient and not sustained *in vivo*. Nonetheless, we hypothesize that CRT may also induce a transient increase in PD-L1 expression *in vivo*, which may only be detectable through mid-treatment biopsies. Consequently, our data suggest a potential benefit in administering immunotherapy in a neoadjuvant setting. Further experimental research is necessary to analyze temporal changes in PD-L1 expression in response to CRT *in vivo* and the feasibility of mid-therapeutic biopsies to thoroughly evaluate this hypothesis. This approach, in conjunction with our findings, could improve the understanding of the optimal timing for immunotherapy in patients with neoadjuvant-treated esophageal cancer.

## Data Availability

The original contributions presented in the study are included in the article/**Supplementary Material**. Further inquiries can be directed to the corresponding author.
