# Supplementary material for: Effects of chemoradiotherapy on surface PD-L1 expression in esophageal cancer and its implications for immunotherapy
Source: Front Immunol. 2024 Dec 23;15:1509051. doi: 10.3389/fimmu.2024.1509051 (PMC11701229; doi:10.3389/fimmu.2024.1509051)
Supplement: Supplementary file 1 [file DataSheet1.pdf]

## Supplementary Material

# 1 Supplementary Figures

## 1.1 Histograms of flow cytometry data

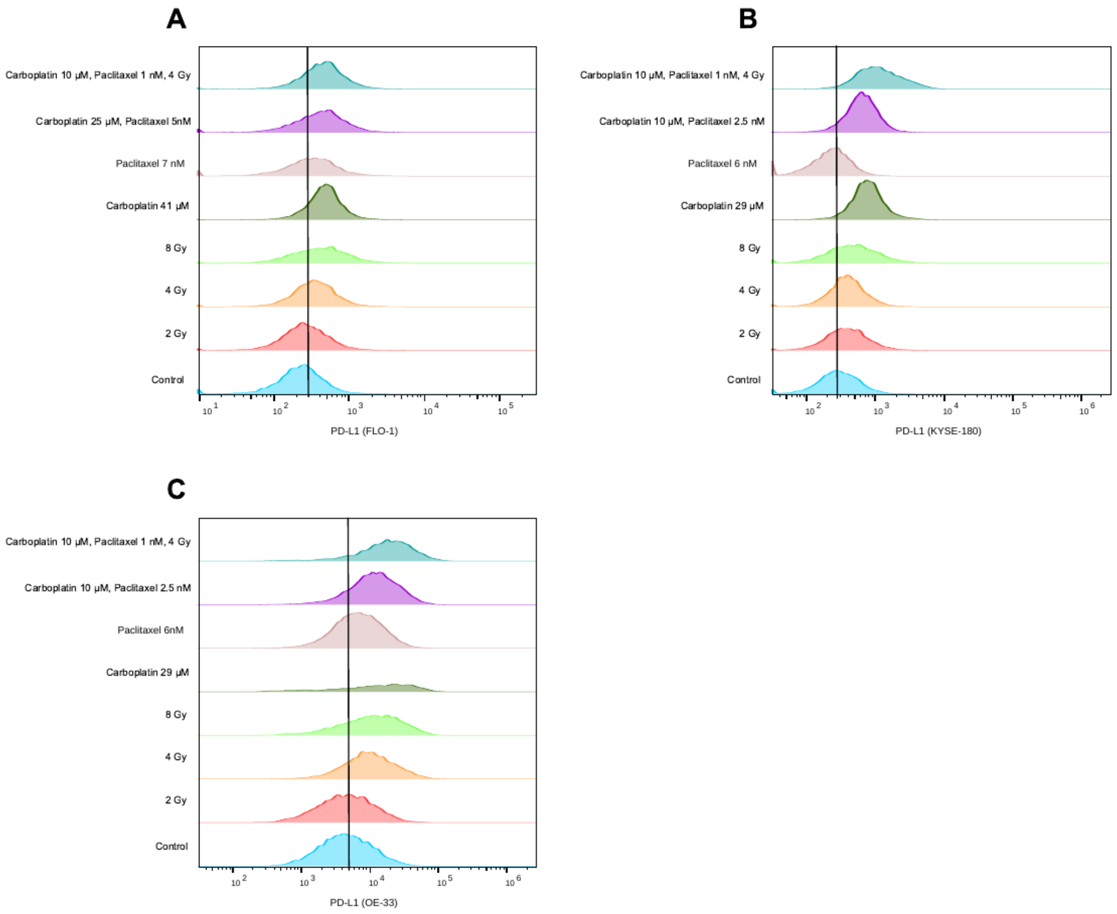


**Supplementary Figure 1 (A-C):** Representative histograms of all treatment conditions in FLO-1 **(A)**, KYSE-180 **(B)** and OE-33 **(C)** and its effects on surface PD-L1. Samples stained with anti-PD-L1-antibody (MIH1) are depicted.

## 1.2 Cell Blocks

### 1.2.1 FLO-1

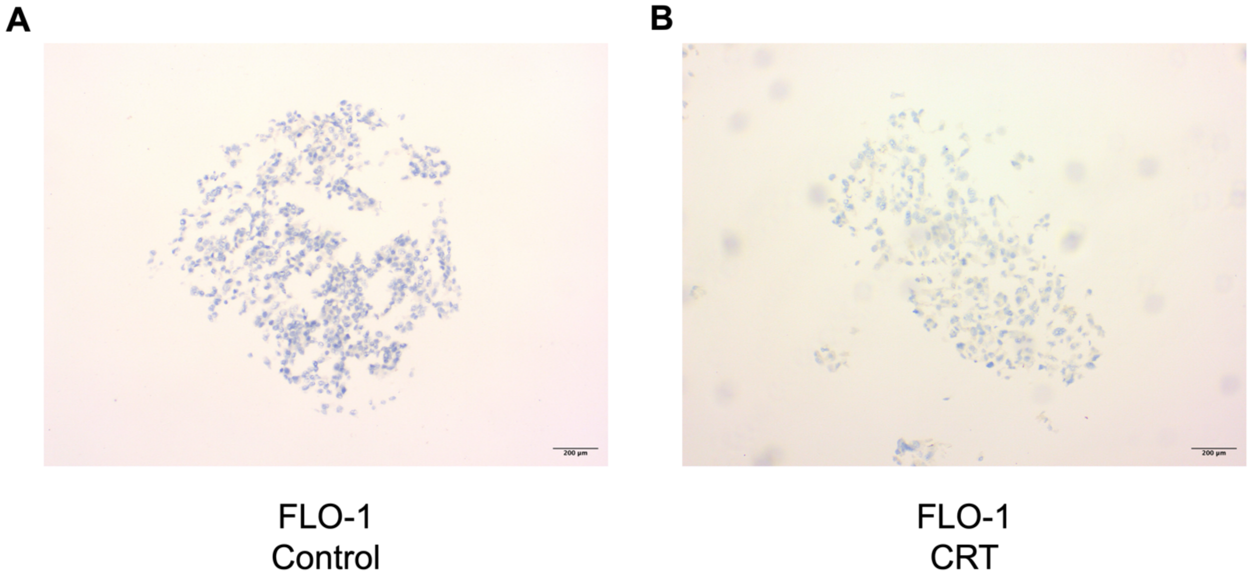


**Supplementary Figure 2:** Cell block of EAC cell line FLO-1 stained with an anti-PD-L1 antibody via immunohistochemistry at 72-hour timepoint **(A-B).** Untreated control **(A)** is depicted next to treated sample **(B)** (Carboplatin 10µM, Paclitaxel 1nm, 4Gy). Images are shown at 10x magnification. CRT = chemoradiotherapy

### 1.2.2 OE-33

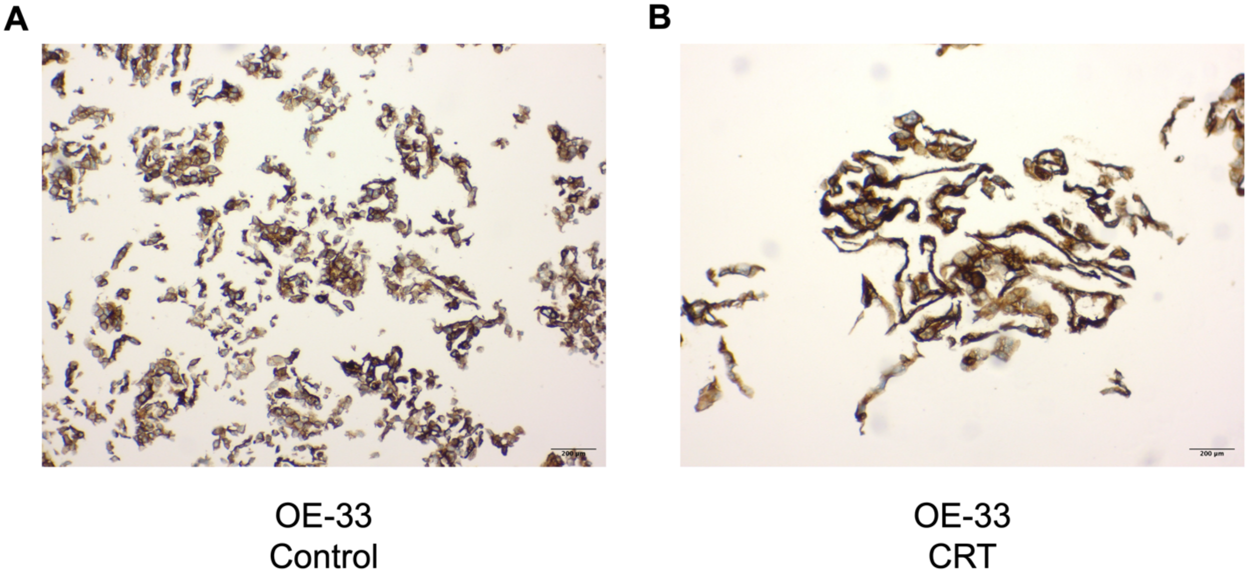


**Supplementary Figure 3:** Cell block of EAC cell line OE-33 stained with an anti-PD-L1 antibody via immunohistochemistry at 72-hour timepoint **(A-B).** Untreated control **(A)** is depicted next to treated sample **(B)** (Carboplatin 10μM, Paclitaxel 1nm, 4Gy). Images are shown at 10x magnification. CRT = chemoradiotherapy

## 1.3 IC-50 Concentrations

### 1.3.1 FLO-1

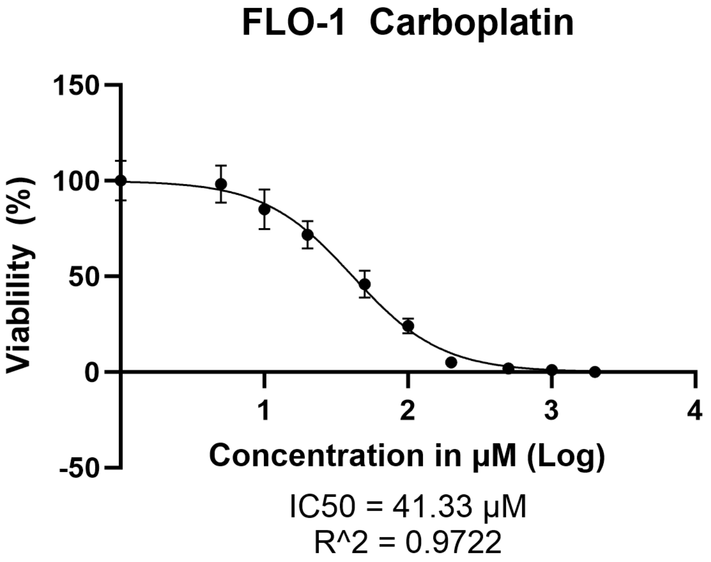


**Supplementary Figure 4:** MTS-Assay determining the IC50 concentration of Carboplatin in EAC cell line cell FLO-1

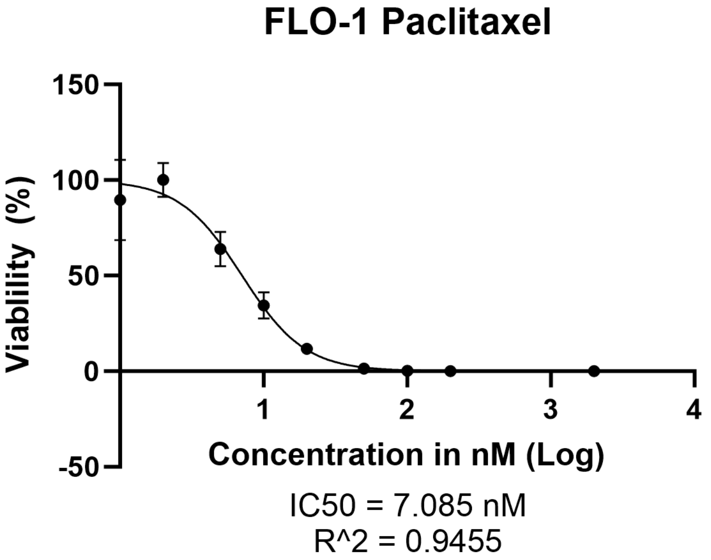


**Supplementary Figure 5:** MTS-Assay determining the IC50 concentration of Paclitaxel in EAC cell line cell FLO-1

### 1.3.2 KYSE-180

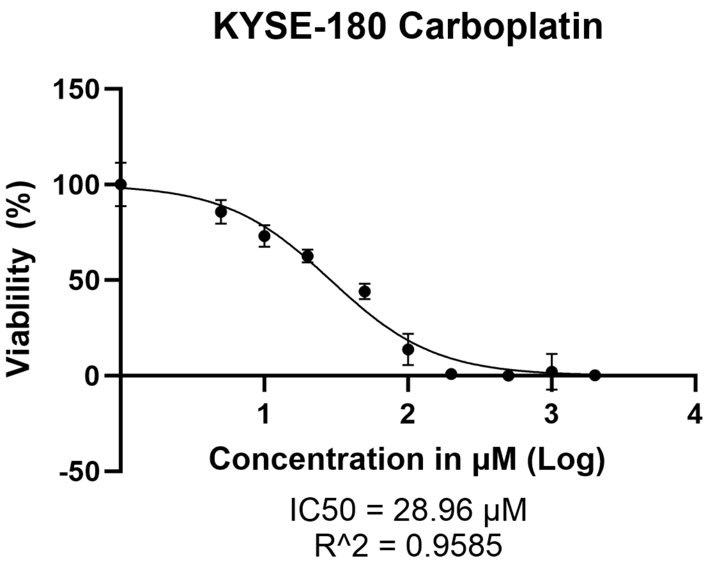


**Supplementary Figure 6:** MTS-Assay determining the IC50 concentration of Carboplatin in ESCC cell line cell KYSE-180

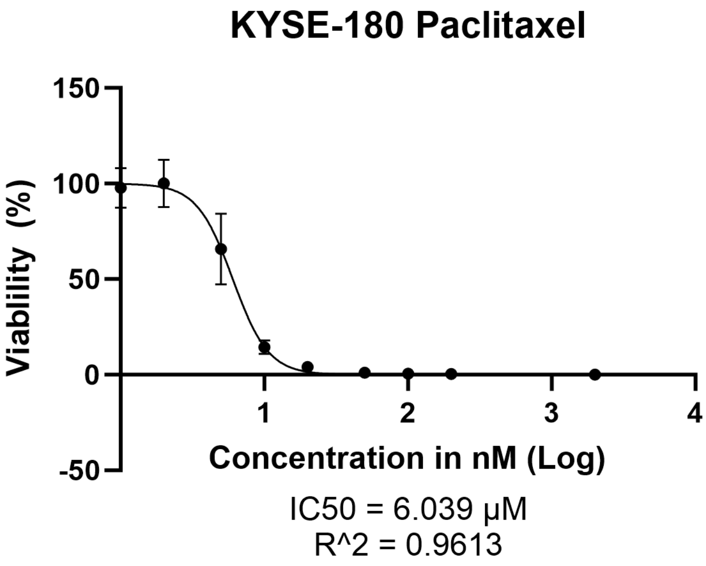


**Supplementary Figure 7:** MTS-Assay determining the IC50 concentration of Paclitaxel in ESCC cell line cell KYSE-180

### 1.3.3 OE-33

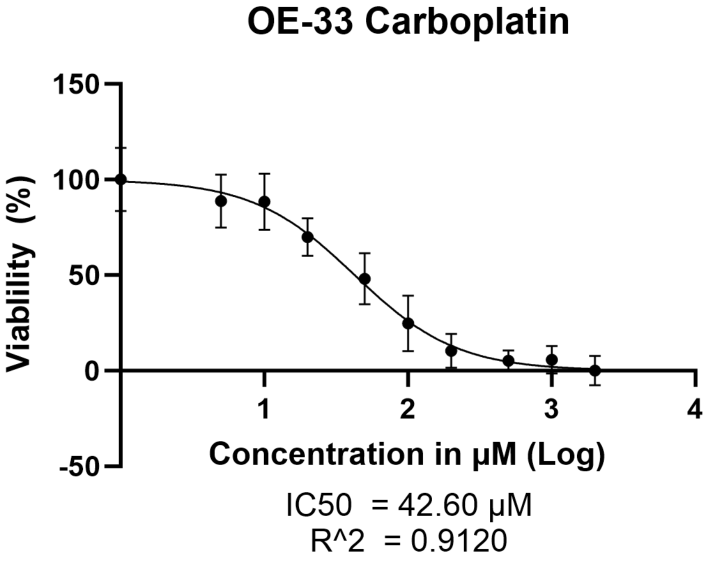


**Supplementary Figure 8:** MTS-Assay determining the IC50 concentration of Carboplatin in EAC cell line cell OE-33

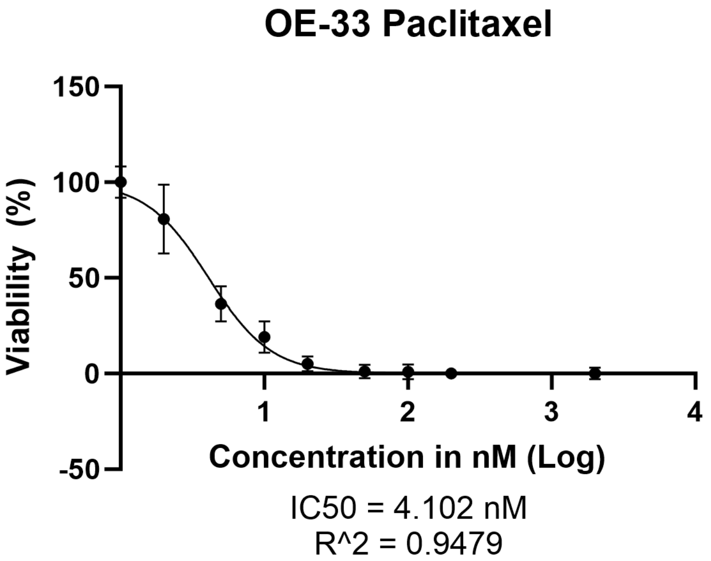


**Supplementary Figure 9:** MTS-Assay determining the IC50 concentration of Paclitaxel in EAC cell line cell OE-33

## 1.4 SynergyFinder

### 1.4.1 FLO-1

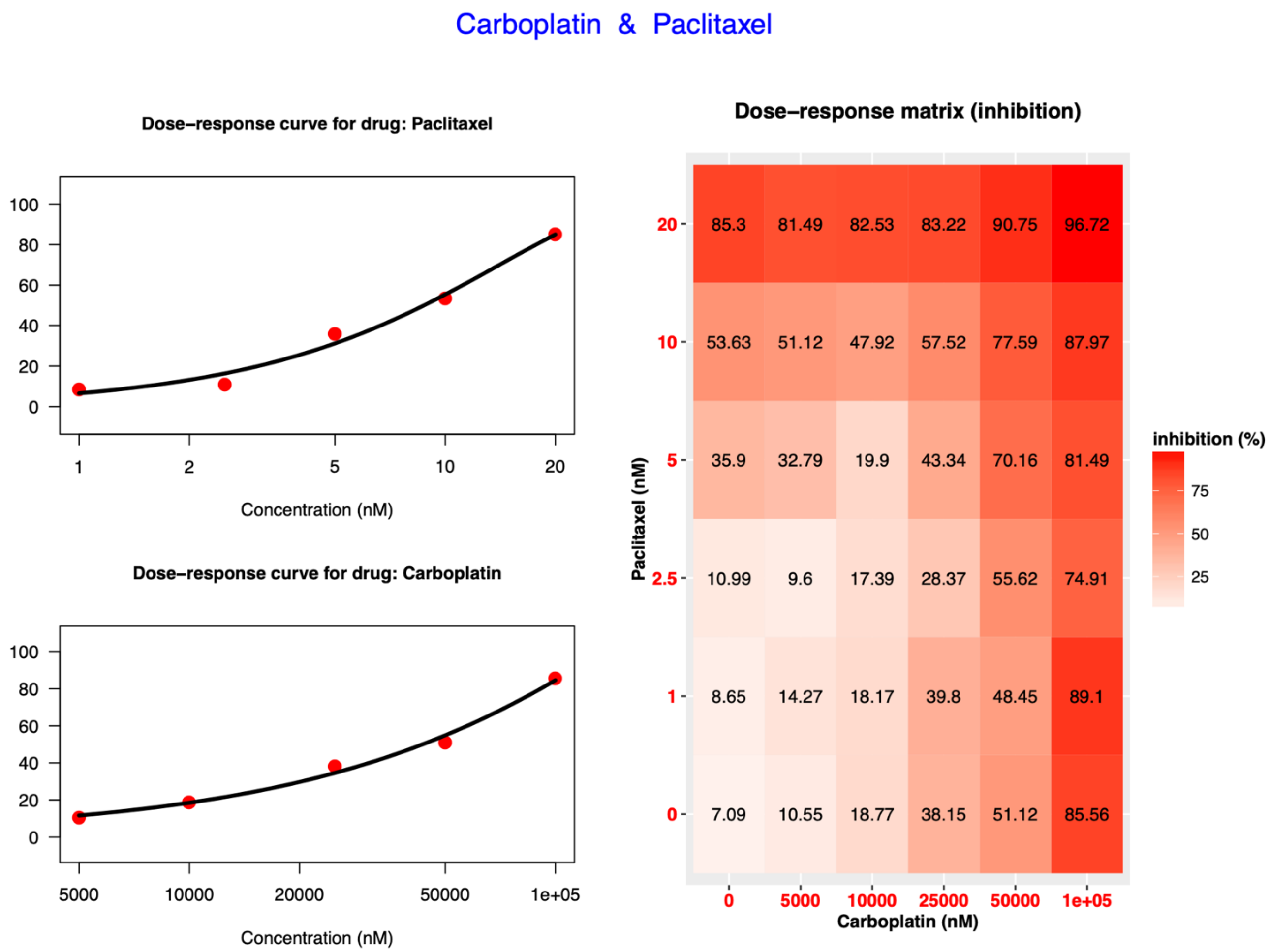


**Supplementary Figure 10:** Synergyfinder output-matrices for combination treatment of Carboplatin and Paclitaxel in EAC cell line FLO-1

### 1.4.2 KYSE-180

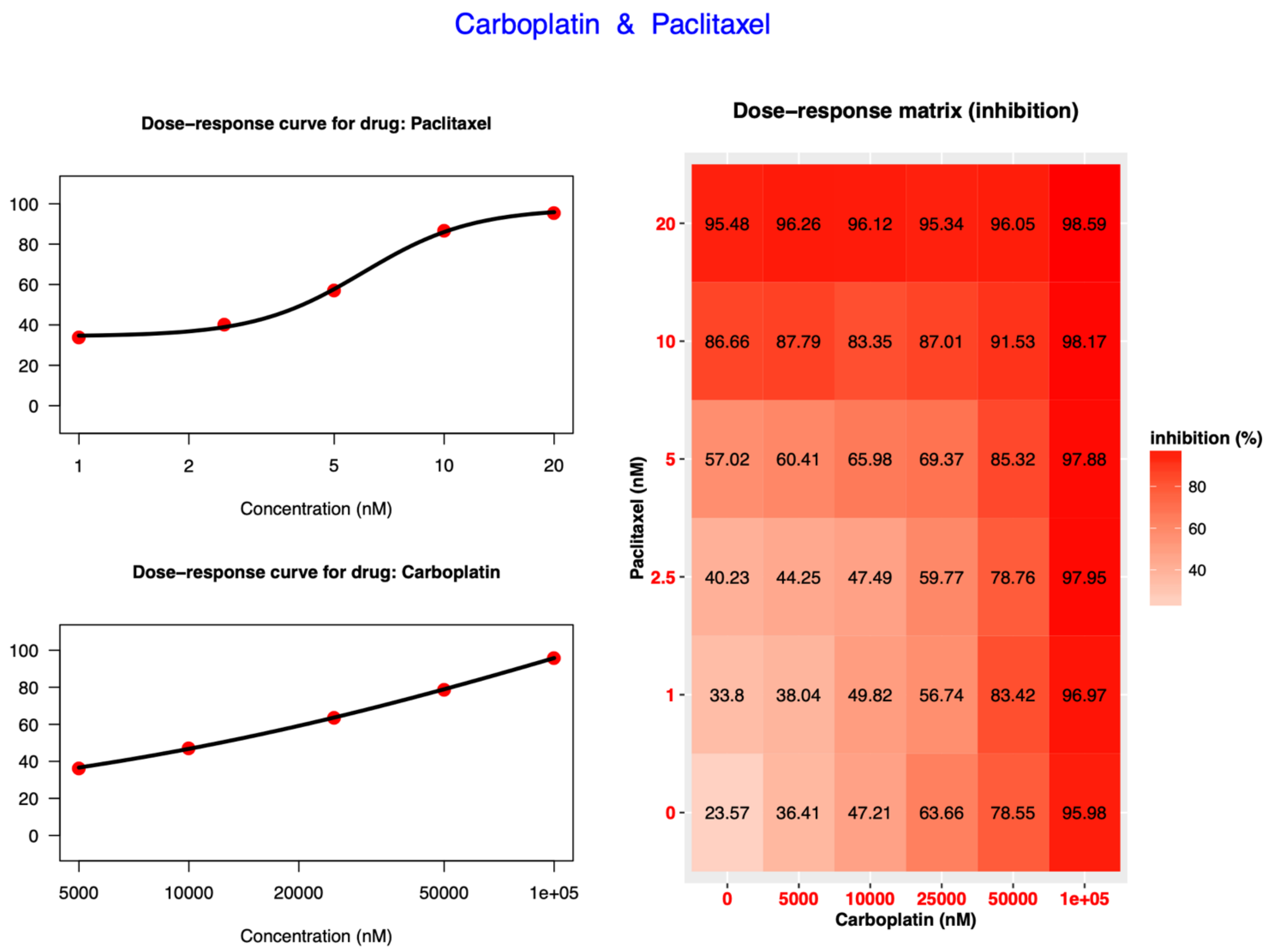


**Supplementary Figure 11:** Synergyfinder output-matrices for combination treatment of Carboplatin and Paclitaxel in ESCC cell line KYSE-180

### 1.4.3 OE-33

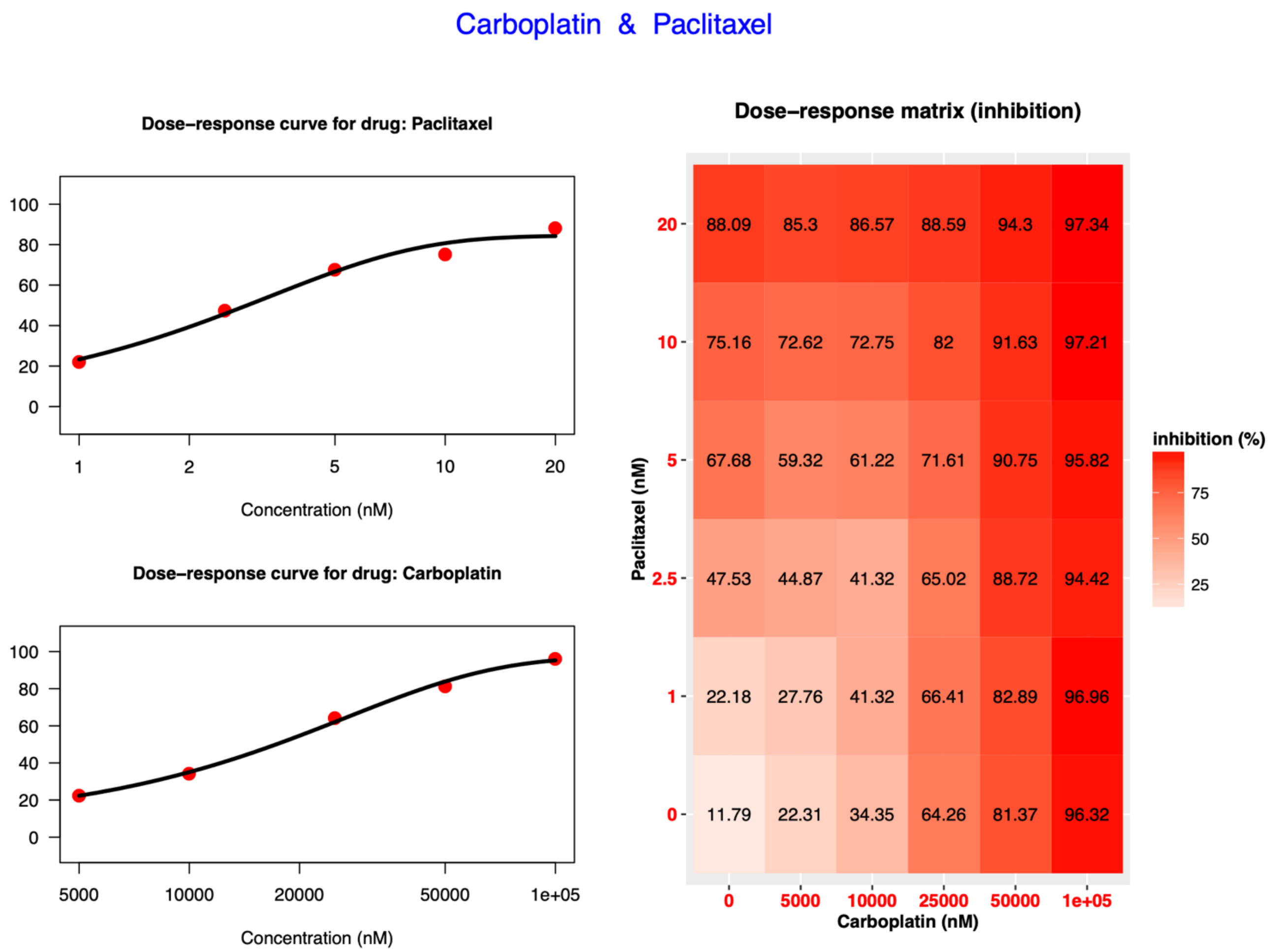


**Supplementary Figure 12:** Synergyfinder output-matrices for combination treatment of Carboplatin and Paclitaxel in EAC cell line OE-33

## 1.5 Gating strategy KYSE-180

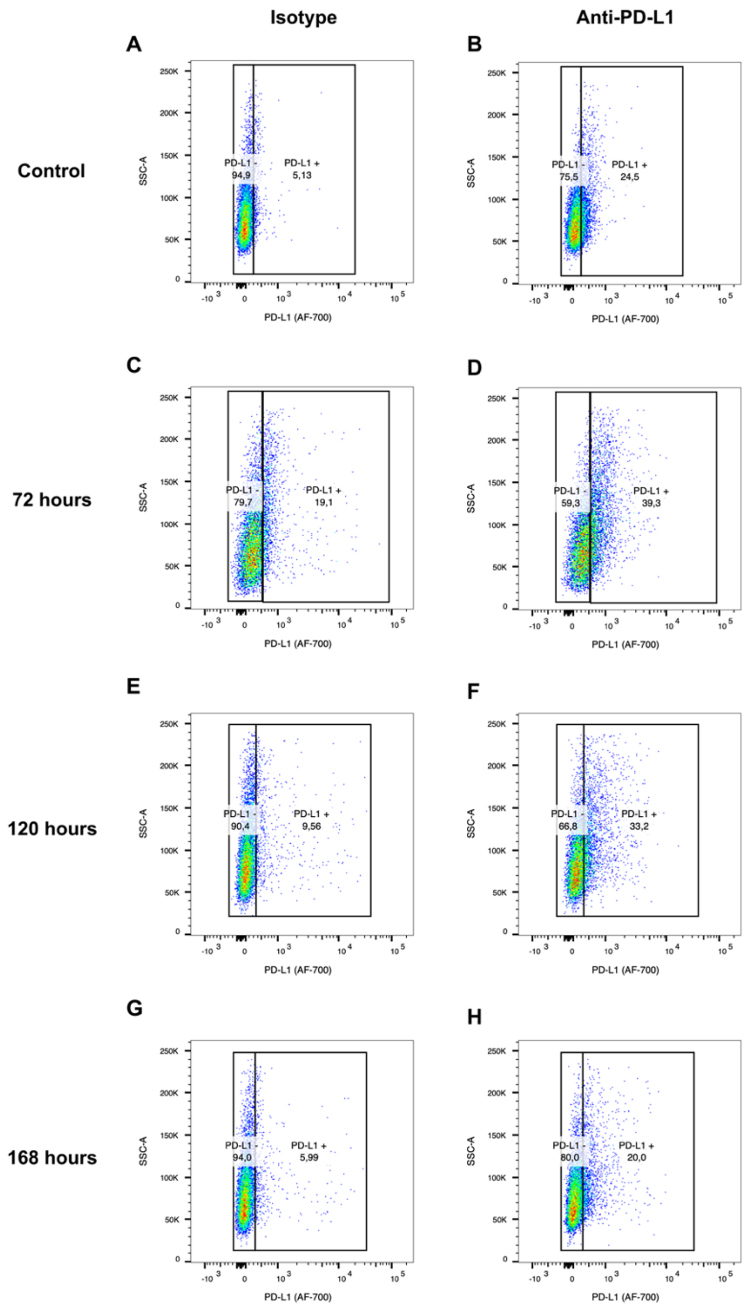


**Supplementary Figure 13:** Gating strategy for the KYSE-180 cell line under control conditions (**A**, **B**) and following treatment with 4 Gy radiotherapy at 72 hours (**C**, **D**), 120 hours (**E**, **F**), and 168 hours (**G**, **H**) post-treatment. Gating was performed based on isotype controls (**A**, **C**, **E**, **G**), with corresponding anti-PD-L1 antibody staining shown in panels **B**, **D**, **F**, and **H**.

## 1.6 List of materials

| Reagent | Catalog number | Manufacturer |
|---|---|---|
| CD274 (PD-L1, B7-H1) Monoclonal Antibody (MIH1), Alexa Fluor™ 700, eBioscience™ | #56-4714-80 | Invitrogen AG |
| Mouse IgG1 kappa Isotype Control (P3.6.2.8.1), Alexa Fluor™ 700, eBioscience™ | #56-4714-80 | Invitrogen AG |
| BD Pharmingen™ Propidium Iodide Staining Solution | 556463 | BD Biosciences |
| RPMI 1640 Medium, GlutaMAX™ Supplement | 61870036 | Gibco™ |
| Fetal+ Triple 0.1µm Sterile Filtered, Fetal Bovine Serum | S-FBSP-EU-015 | Serana Europe GmbH |
| Trypsin-EDTA | 15400054 | Gibco™ |
| Titriplex® III | 108418.0250 | Merck KGaA |
| Dulbecco's Phosphate-Buffered Saline | L1820 | Biochrom GmbH |
| Dimethyl Sulfoxide | 67-68-5 | Sigma-Aldrich Corp. |
| Carboplatin | J60433.03 | Thermo Fisher Scientific Chemicals |
| Paclitaxel +99 % | 328420050 | Thermo Fisher Scientific Chemicals |
| CellTiter 96® AQueous One Solution Cell Proliferation Assay (MTS) | G3582 | Promega Corp. |
| OE-33 cell line | ACC 706 | DSMZ-German Collection of Microorganisms and Cell Cultures GmbH |
| FLO-1 cell line | ACC 698 | DSMZ-German Collection of Microorganisms and Cell Cultures GmbH |
| KYSE-180 cell line | ACC 379 | DSMZ-German Collection of Microorganisms and Cell Cultures GmbH |
| EnVision Flex Target Retrieval Solution | K800521-2 | Agilent Technologies, Inc. |
| PD-L1 IHC 22C3 pharmDx (Dako Omnis) | GE006 | Agilent Technologies, Inc. |
| EnVision Flex+, Dako Agilent | GV821 | Agilent Technologies, Inc. |
| Mayers Hämatoxylin | MHS32 | Sigma-Aldrich Corp. |

**Supplementary Figure 14:** Comprehensive list of materials, including catalog numbers and manufacturers, for all reagents utilized in this manuscript.
